# Molecular mechanisms of ionic liquid cytotoxicity probed by an integrated experimental and computational approach

**DOI:** 10.1038/srep19889

**Published:** 2016-02-02

**Authors:** Brian Yoo, Benxin Jing, Stuart E. Jones, Gary A. Lamberti, Yingxi Zhu, Jindal K. Shah, Edward J. Maginn

**Affiliations:** 1 University of Notre Dame, Department of Chemical and Biomolecular Engineering, Notre Dame, IN 46556-5637, USA; 2 University of Notre Dame, Department of Biological Sciences, Notre Dame, IN 46556-0369, USA; 3 Wayne State University, Department of Chemical Engineering and Materials Science, Detroit, MI 46202 USA; 4 Oklahoma State University, School of Chemical Engineering, Stillwater, OK 74078, USA

## Abstract

Ionic liquids (ILs) are salts that remain liquid down to low temperatures, and sometimes well below room temperature. ILs have been called “green solvents” because of their extraordinarily low vapor pressure and excellent solvation power, but ecotoxicology studies have shown that some ILs exhibit greater toxicity than traditional solvents. A fundamental understanding of the molecular mechanisms responsible for IL toxicity remains elusive. Here we show that one mode of IL toxicity on unicellular organisms is driven by swelling of the cell membrane. Cytotoxicity assays, confocal laser scanning microscopy, and molecular simulations reveal that IL cations nucleate morphological defects in the microbial cell membrane at concentrations near the half maximal effective concentration (EC50) of several microorganisms. Cytotoxicity increases with increasing alkyl chain length of the cation due to the ability of the longer alkyl chain to more easily embed in, and ultimately disrupt, the cell membrane.

Unlike conventional alkali halide salts, ionic liquids (ILs) are liquid at moderate temperatures. Properties including negligible vapor pressure, ability to dissolve both polar and nonpolar species, and excellent electrochemical stability have generated immense interest in these novel liquids for a variety of commercial applications^1^,^2^. Although the very low volatility of ILs implies that fugitive gas phase emissions are negligible, ILs typically exhibit significant solubility in water, suggesting that the most likely pathway for release of ILs into the environment is via aqueous waste streams^3^. Once discharged into the environment, they are likely to persist in water and soil due to their high solubility and variable biodegradability^4^,^5^,^6^. Moreover, ILs can induce cytotoxicity among a wide range of organisms^7^. Thus, the design of environmentally friendly ILs must include a complete life-cycle assessment including toxicity, biodegradability, and eventual fate and transport of these novel solvents.

Although a number of studies have reported the species-specific toxic effects of ILs^8^,^9^,^10^, a comprehensive understanding of the molecular mechanisms by which ILs are rendered toxic is still lacking. In addition to designing inherently safe ILs, such knowledge is vital in many biological applications where ILs are being investigated as antimicrobial, antifungal, and therapeutic agents^11^,^12^. New information on IL-cell interactions will also play a critical role in designing novel bioprocesses based on ILs^13^. Here we integrate toxicology and biophysical experiments with atomistic and coarse grained (CG) molecular dynamics simulations to elucidate the molecular mechanisms by which the toxicity of ILs of the popular class 1-*n*- alkyl-3-methylimidazolium ([C_*n*_mim]^+^, *n* = 4 to 12) in combination with Cl^−^ increases with alkyl chain length.

## Results

### Cytotoxicity studies

**Figure 1:**
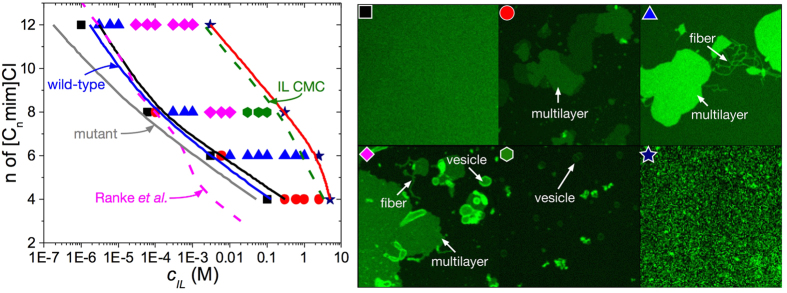
Phase diagram of ionic liquid induced cytotoxicity. [C_*n*_mim]Cl induces morphological changes to a supported *α*-PC bilayer. The dashed magenta line in the phase diagram corresponds to the response of EC_50_ to ionic liquid alkyl chain length for IPC-cell lines from Ranke *et al*.^14^. Similarly, the solid blue and grey lines depict predicted EC_50_ as a function of ionic liquid alkyl chain length for wild-type (with cell wall) and mutant (without cell wall) strains of *Chlamydomoas reinhardtii*, respectively. See Ecotoxicity Assay Details for further information about how these relationships were generated. The dashed green line corresponds to the IL critical micelle concentration (CMC) reported by Blesic *et al*.^19^ at similar ionic strengths to the confocal microscopy experiments (CMC for [C_4_mim]Cl has been extrapolated). Colored symbols in the figure correspond to the specific lipid morphology as shown in the right image: black square - featureless supported lipid bilayer; red circle - multilayer; blue triangle - multilayer and fiber/tube; pink diamond - multilayer, fiber/tube and vesicle; green hexagon - vesicle; and navy star - disrupted bilayer captured at much higher detector gain to improve the image quality. The size of all the micrographs is 30 *μ*m by 30 *μ*m and represents a top-down view of a fluorescently labeled lipid bilayer adsorbed onto a solid support. The solid black and red lines correspond to the onset of supported lipid bilayer disruption and the total disruption of the supported lipid bilayer, respectively.

Cytotoxicity studies were performed on two strains of a freshwater green alga (*Chlamydomonas reinhardtii*) - a wild-type strain possessing a cell wall and a mutant strain lacking a cell wall - to observe any modifications in the IL toxicity for organisms with or without a cell wall. The half maximal effective concentrations (EC_50_) were estimated (Fig. 1) and are compared with previous results for mammalian IPC-cell lines from Ranke *et al*.^14^. Surprisingly, we find that the EC_**50**_ data overlaps with the onset IL concentration for morphological changes of a lipid bilayer (see below). The presence of a cell wall increased the EC_50_, perhaps by reducing the dispersive effect of ionic liquid moieties on lipid bilayers. In all cases it was observed that the EC_50_ decreased (i.e. toxicity increased) as the alkyl chain length *n* on the cation increased, consistent with the known observation that longer alkyl chains lead to greater toxicity^15^,^16^.

### Biophysical microscopy studies

Biophysical experiments with confocal laser scanning microscopy (CLSM) were used to study the interaction of aqueous ILs with a lipid bilayer, a commonly used model for a cell membrane. While this model system obviously lacks many of the typical transmembrane entities found in actual cells, it can probe morphological effects to the lipid bilayer which are also likely to significantly alter the functionality of such entities. As shown in Fig. 1, a bare supported L-α-phosphatidylcholine (α-PC) bilayer consisting of 1 mol% fluorescent lipids appears homogeneous and featureless, consistent with previous work^17^. Shortly after adding aqueous solutions of ILs, many aggregates appeared in the form of disks, multilayers, fibers, and vesicles. Critical IL concentrations at which this morphological reorganization commences decreased significantly with increasing alkyl chain length of the cation, and also nearly overlap with the EC_50_ concentrations measured for wild-type and mutant strains of *C. reinhardtii* as well as those for mammalian IPC-cell lines^14^. These results collectively suggest that morphological restructuring of lipid bilayers induced by the IL is linked to the cytotoxicity of aqueous solutions of ILs.

To test whether ILs insert into the lipid bilayer, fluorescence intensity measurements of a membrane fusion probe (R18) mixed with the *α*-PC bilayer were carried out (see Supplementary Information, Fig. S1). Due to a self-quenching effect, the fluorescence intensity of the probe increases upon dilution. The fluorescence intensity of R18 increased with time once ILs were introduced in the system, suggesting that the ILs insert into the bilayer and that incorporation of cations into the lipid bilayer is energetically favored for the reassembly of lipids into the observed morphologies. Considering the surfactant-like nature of ILs and that they are the sole additives in the system, their tendency to insert into the bilayer is not unexpected, as common surfactants are known to exhibit similar behavior^18^. The upper concentrations at which the bilayer shows complete disintegration (Fig. 1) closely overlap with the reported critical micelle concentration (CMC) of the ILs^19^. Taken together, these observations suggest that the molecular mechanism of IL cytotoxicity may be linked to the IL-induced morphological reorganization of cell membranes initially caused by the insertion of ILs into the membrane. We caution, however, that additional work with more realistic systems is needed to definitively prove this mechanism.

### Molecular simulation studies

To explore the molecular-level details of IL insertion into the lipid bilayer in greater detail, molecular dynamics (MD) simulations were carried out for a 1-palmiltoyl-2-oleoyl-phosphatidylcholine (POPC) lipid bilayer (T_*m*_ = −2 °C) system in contact with aqueous imidazolium-based ionic liquid solutions. Note that POPC is the most abundant lipid in *α*-PC. The concentrations examined in the simulations are comparable to those used in the fluorescence microscopy and cytotoxicity experiments and are below the experimental solubility limits for these ILs^20^,^21^. Simulation details are provided in the Supplementary Information.

**Figure 2:**
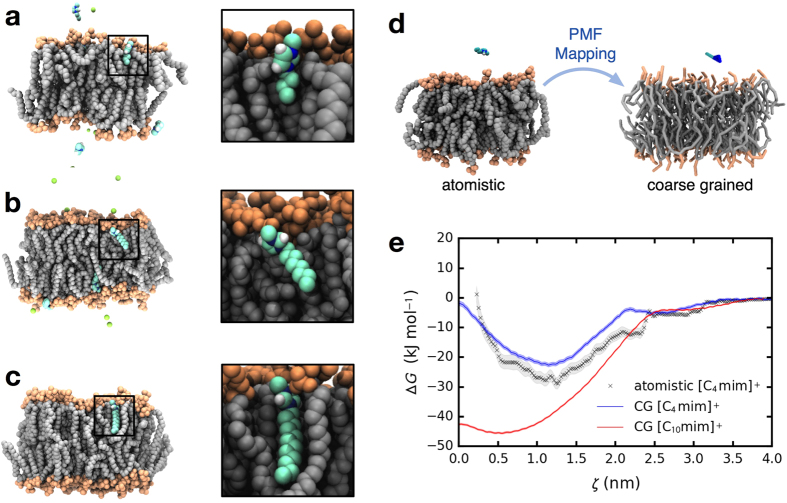
Simulating insertion of ionic liquid into a lipid bilayer. (**a–c**) Atomistic simulations of a POPC bilayer system with [C_4_mim]Cl, [C_8_mim]Cl, and [C_12_mim]Cl demonstrating spontaneous insertion of imidazolium cations into the lipid bilayer. IL concentrations ranged from ~5–50 mM. (**d**) Schematic diagram of the PMF mapping operator between the atomistic and coarse grained models. The PMF mapping method was used to obtain refined IL and lipid cross interaction parameters for the coarse grained models. **(e),** PMF or transfer free energy of a single IL cation insertion into a lipid bilayer for both atomistic and coarse grained simulations. The cross interaction parameters have been transferred to longer alkyl side chain cations (e.g. [C_10_mim]^+^).

We previously used atomistic MD simulations^22^ to investigate the interactions between imidazolium-based ILs with cations having varying alkyl chain lengths 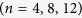 and a POPC bilayer. This work was motivated by studies^14^,^23^ showing that IL ecotoxicity is intimately linked to the enhanced lypophilic nature of ILs. The simulations suggest that [C_*n*_mim]^+^ cations tend to insert spontaneously into the lipid bilayer (Fig. 2a–c), with the alkyl tails clearly embedding into the bilayer. Further, the longer the alkyl chain, the deeper the penetration into the bilayer.

Atomistic simulations of this type are limited in the time and length scales that can be accessed, and thus are unable to model the swelling observed in the microscopy experiments. To extend the time and length scales of the simulations, a coarse grained model was parameterized against the results of the atomistic simulations. Free energy profiles (or potentials of mean force, PMFs) for insertion of a coarse-grained model of [C_4_mim] were computed and compared against PMFs calculated using the fully atomistic representation of the IL. The coarse grained model parameters were adjusted to match the atomistic PMF and the parameters were then extended to [C_10_mim]. Figure 2(d,e) show the mapping procedure and that the coarse grained model PMF matches that of the atomistic system. Details of the parameterization procedure and the coarse grained force field parameters are given in Supporting Information.

**Figure 3:**
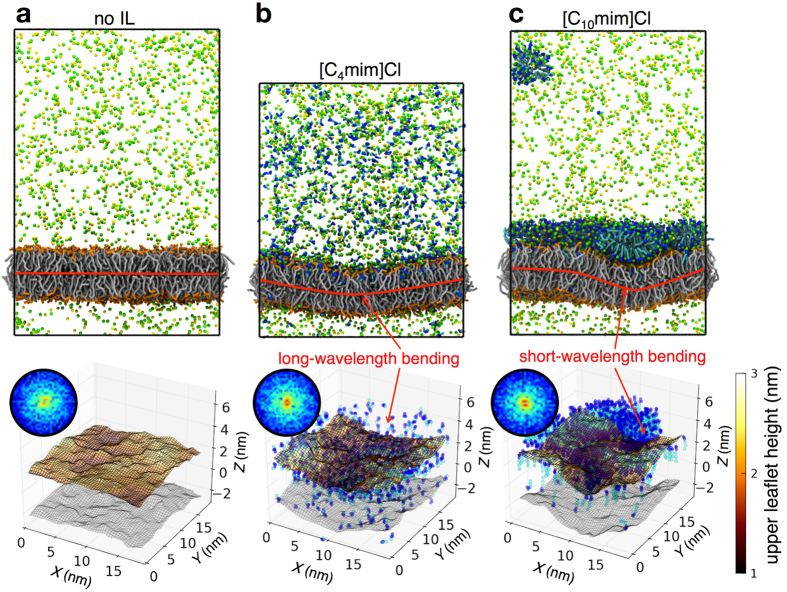
Ionic liquids induce bending of the lipid bilayer. (**a**–**c**) Snapshots of coarse grained simulations of a POPC bilayer system (**a**) without ILs, (**b**) with [C_4_mim]Cl and (**c**) with [C_10_mim]Cl (top row). IL and NaCl (sodium-yellow; chloride-green) buffer concentrations are ~200 mM and ~160 mM respectively. In (**c**), a nearly fully covered monolayer of adsorbed [C_10_mim]Cl is formed at the lipid bilayer aqueous interface. Coarse grained water molecules are not displayed. Interactions between IL cations with the POPC bilayer induce bending of the bilayer. The bilayer center of mass plane is shown in red to guide the eye. 3-D contour surface plots of the upper leaflet height based on the phosphate group of the lipid are plotted (bottom row). The lower leaflet surface is shown in gray. IL cations are only shown for clarity (imidazolium ring - blue; alkyl side chain - cyan). Z-coordinates have been recentered to the bilayer center plane. Spectral intensity based on the Fourier transform of the bilayer surface along the X and Y plane (bottom row inset). Intensity peaks corresponding to the bright red regions are associated with high amplitude bending modes of the bilayer surface.

Using the coarse grained models, simulations of [C_*n*_mim]Cl, with *n* set to 4 and 10, were performed for large system sizes near the continuum limit. To model a scenario more typical of the microscopy experiments, the majority of the ILs at concentrations of ~200 mM were initially placed on one side of the bilayer along with Na^+^ and Cl^−^ ions consistent with a 160 mM NaCl buffer. Although the simulated concentration was above the CMC for [C_10_mim]Cl, the same concentration was used to maintain a high ratio of ILs to lipids. As expected, both short (*n* = 4) and long (*n* = 10) alkyl side chain cations spontaneously inserted into the lipid bilayer with the same orientation as that in the atomistic simulations (Fig. 3). During the course of the [C_4_mim]Cl simulations, the number of inserted cations into the upper bilayer leaflet reached a saturation limit of approximately 0.6 inserted cations per lipid in a single bilayer leaflet. Within a few ns of simulation time after saturation, the bilayer began to undergo long-wavelength bending in response to the asymmetric distribution of inserted cations, as indicated by the bright red regions in the spectral intensity of the bilayer undulation surface (Fig. 3). Consistent with this, the bending modulus dropped from 22.6 ± 1.7 × 10^−20^ J for a system without any ILs to 9.3 ± 0.9 × 10^−20^ J with ILs. Similar bending modes were not observed after inserted cations finally saturated both the upper and lower leaflets of the bilayer. This suggests that the observed large bending fluctuations can be a direct consequence of asymmetric insertion of ILs and the inability for inserted ILs to transfer across the hydrophobic center of the bilayer to the opposing leaflet. Interestingly, for the long chain (*n* = 10) cation system, although some cations spontaneously inserted into the lipid bilayer, the majority of the cations spontaneously self-assembled into micelles, eventually adsorbing onto the upper bilayer leaflet surface to form an IL monolayer. Likewise for this system, inserted or micelle-adsorbed ILs did not transfer across bilayer leaflets. The adsorbed ILs also induced bending of the bilayer, although the bending was much more localized and highly correlated with the location of the IL monolayer. In addition, the bending modulus dropped to 8.9 ± 0.8 × 10^−20^ J. Within the simulated timescale, no fusion of IL monolayer into the lipid bilayer or desorption of the IL monolayer from the upper bilayer leaflet interface was observed.

For both atomistic and coarse grained simulations, no evidence was found of deep insertion of Cl^−^ anions into the lipid bilayer hydrophobic center, implying that the hydrophobic interaction between the lipid and the alkyl chain was the primary driving force for partitioning of the IL cation into the lipid bilayer.

## Discussion

Results of toxicity studies, confocal microscopy experiments, and molecular simulations suggest a mechanism for ionic liquid toxicity that involves cation insertion into the cell membrane. Our results are consistent with a number of previous reports indirectly pointing to such a mechanistic pathway^24^,^25^,^25^,^26^,^27^,^28^,^29^,^30^. Our work elaborates on this pathway to show that as the number of inserted cations reaches a saturation limit, the bilayer buckles to maximize the surface area for bilayer and IL hydrophobic interactions. Such large scale buckling modes of the bilayer seems to be a direct consequence of the asymmetric insertion of ILs into a bilayer leaflet and the inability for ILs to transfer across the bilayer after being inserted, although we plan to address such effects more closely in our future work. For longer chain cations, the cytotoxicity is further enhanced due to stronger IL-lipid bilayer interactions. We propose that the IL-induced bending instabilities can subsequently cause morphological reorganization of the cell membrane. Such significant morphological effects will likely alter cell functionality, some of which may be vital to cells (e.g. conformational changes to transmembrane protein, small molecule membrane permeability, lipid lateral mobility and reconstruction capability), ultimately leading to cell death.

## Methods

### Ecotoxicity Assay Details

Two strains of a freshwater green alga (*Chlamydomonas reinhardtii*), namely a wild-type strain possessing a cell wall (CPCC *#*243) and a mutant strain lacking a cell wall (CPCC *#*12), were obtained from the Canadian Phycological Culture Centre (CPCC). Stocks were maintained in a modified high salts medium (HSM)^31^ at room temperature on a 12-h cycle of artificial light.

Imidazolium-based ionic liquids (IOLITEC Inc.; Tuscaloosa, AL) consisted of three different cations paired with a Cl^−^ anion. The cations differed in their alkyl chain lengths (4, 8, and 12), and were in the form of 1-butyl-, 1-octyl-, and 1-dodecyl-3-methylimidazolium.

For all growth experiments, four replicate samples of 4 to 10 concentrations of ILs and an IL-free control were used. *C. reinhardtii* was grown in 500-mL Erlenmeyer flasks containing 125 mL of HSM. Each replicate was inoculated with 1 mL of stock culture and incubated at room temperature on an orbital shaker table under artificial growth lights (12-h light-dark cycle). To determine growth, chlorophyll a from each replicate culture was measured daily using *in vivo* fluorescence on a Turner Trilogy fluorometer^32^.

Growth rates were calculated for each experimental culture using the spline method of the grofit package^33^ implemented in the R Statistics Package^34^. Using growth rate and IL concentration data, the half maximal effective concentrations (EC_50_) and their standard errors were estimated from the better of two model fits (general logistic model and an extended logistic model, which allows for increased growth at low toxin levels or hormesis) using maximum likelihood methodologies^35^,^36^. Ninety-five percent confidence intervals for the EC_50_ concentrations were estimated using the Hessian of the maximum likelihood fits provided by the optim function in the R Statics Environment^34^. The effect of a cell wall on IL toxicity was inferred from differences in EC_50_ between the two *C. reinhardtii* strains, and statistical significance was inferred from a lack of overlap of 95% confidence intervals of EC_50_ estimates for the two strains. The relationship between EC_50_50 and the alkyl chain length of the ionic liquid was determined using linear regression.

### Biophysical Study Details

The compounds *α*-PC and fluorescent 1,2-dioleoyl-sn-glycero-3-phosphoethanolamine-N-(lissamine rhodamine B sulfonyl) ammonium (LR-PE), both purchased from Avanti Polar Lipids, were used to produce liposome and supported lipid bilayers (SLBs) on polished quartz coverslips (ESCO Products). Quartz coverslips were cleaned by sonication in ethanol for 10 min and then were soaked in a heated piranha solution (30% H_2_O_2_ and 70% H_2_SO_4_) at 110 °C for 1 h. Subsequently, quartz coverslips were thoroughly rinsed with deionized water (Barnstead Nanopure II) and dried with nitrogen gas (purity >99.9%) before use. The membrane fusion probe, octadecyl rhodamine B Chloride (R18) was purchased from Life Technologies and used directly. Phosphate-buffered saline (PBS) was purchased from VWR and used directly. Imidazolium-based ionic liquids (IOLITEC Inc.; Tuscaloosa, AL) used in this study possess a Cl^−^ anion, while cations differ in their alkyl chain lengths (4, 6, 8, 12) in the form of 1-butyl-, 1-hexyl-, 1-octyl-, and 1-dodecyl-3-methylimidazolium.

Small unilamellar vesicles (SUVs) were prepared by the commonly used extrusion method^17^. Briefly, a mixture of *α*-PC with fluorescence dye in chloroform was dried by nitrogen gas to form a dry mixed lipid film and then re-suspended in PBS to form lipid vesicles with a final concentration of 1.0 g/L via sonication for 10 min before and after storage at T = −25 °C overnight. Subsequently, the suspension of hydrated lipid vesicles was extruded repeatedly through a mini-extruder (Avanti Polar Lipids) with two layers of polycarbonate membrane filters of 100 nm pore diameter (Whatman; Maidstone, UK) to yield SUVs of diameter (*d*) ~120 nm as determined by dynamic light scattering (Brookhaven Instruments). For all fluorescence microscopy experiments, the molar ratio of *α*-PC to fluorescence dye in the mixed SLBs was kept constant at 100:1. The SLB was prepared by SUV fusion and disruption method, where 1 mL of 1.0 mg/mL SUV suspension in PBS was added to a cleaned quartz coverslip and kept incubated in a custom-built liquid cell for 30 min to obtain a SLB. Excess SUVs were removed by repeatedly and gently rinsing the SLB with deionized water, and then incubated in PBS buffer for 30 min before use. The SLB prepared by this method was homogenous and featureless, as verified in our previous work^17^,^37^,^38^. The morphology of SLBs with/without added ILs was characterized in real time by confocal laser scanning microscope (CLSM, Zeiss LSM 5 Pascal) with a 100X objective lens (NA = 1.4, oil immersion) and analyzed by ImageJ^39^.

### Simulation Details

Coarse grained simulations and potential of mean force (PMF) calculations were carried out using Gromacs 4.5.5^40^,^41^,^42^. The SDK models^43^,^44^,^45^ were used to simulate the POPC lipid bilayer in sodium chloride buffer solution, while the IL model was taken from Bhargava and Klein^46^ and Bhargava *et al*.^47^. For all of the models used in this work, the interaction potential was described by a 9–6 Mie potential for pairs excluding water or by a 12–4 Mie potential pairs including water. All simulations were performed in the semi-isotropic NPT ensemble for 250 ns and with a dielectric constant 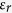 set to 16. The procedure for developing new coarse grained interaction parameters is described in Supplementary Information.

## Additional Information

**How to cite this article**: Yoo, B. *et al*. Molecular mechanisms of ionic liquid cytotoxicity probed by an integrated experimental and computational approach. *Sci. Rep.* **6**, 19889; doi: 10.1038/srep19889 (2016).

## Supplementary Material

Supplementary Information
