# Supplementary material for: Molecular mechanisms of ionic liquid cytotoxicity probed by an integrated experimental and computational approach
Source: Sci Rep. 2016 Feb 2;6:19889. doi: 10.1038/srep19889 (PMC4735680; doi:10.1038/srep19889)
Supplement: Supplementary Information [file srep19889-s1.pdf]

Supplementary Information: Molecular mechanisms of ionic liquid cytotoxicity probed by an integrated experimental and computational approach

Brian Yoo[1], Benxin Jing[1], Stuart Jones[2], Gary Lamberti[2],

Yingxi Zhu[1], Jindal K. Shah[3], Edward J. Maginn[1]*

University of Notre Dame

[1]*Department of Chemical and Biomolecular Engineering*

University of Notre Dame, 182 Fitzpatrick Hall, Notre Dame, IN 46556-5637, USA

[2]*Department of Biological Sciences*

University of Notre Dame, Notre Dame, IN 46556-5637, USA

[3]*School of Chemical Engineering*

Current address: Oklahoma State University, Stillwater, OK 74078, USA

email: glambert@nd.edu, yzhu3@nd.edu, jindal.shah@okstate.edu, ed@nd.edu

Fluorescence intensity measurements

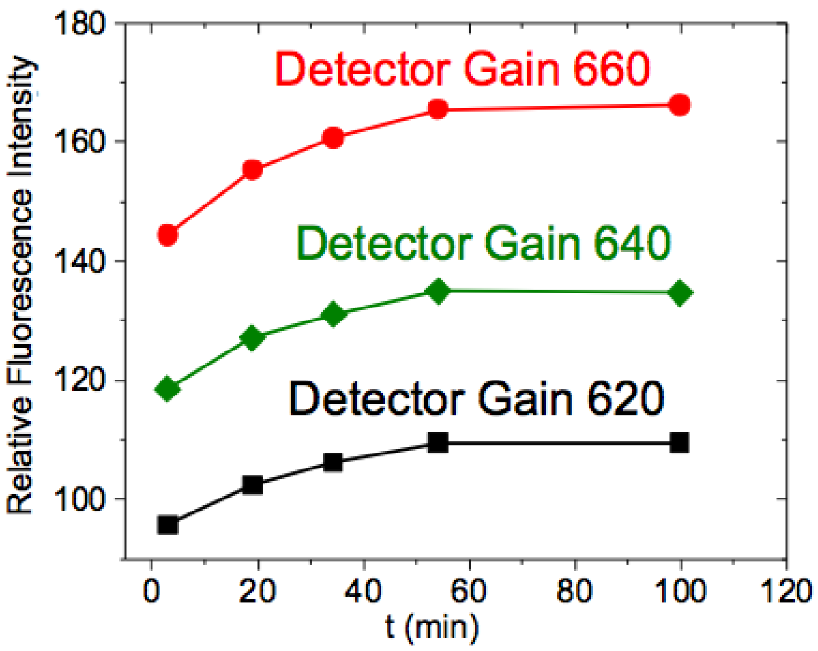

Figure S1 Fluorescence probe of ionic liquid insertion into the lipid bilayer. Time evolution of a 4 mol% R18 fluorescence intensity in the α-PC supported bilayer after adding 10 mM [C_8mim]Cl. Fluorescent intensities have been measured in the CLSM at different detector gains: 620 (square), 640 (diamond) and 660 (sphere).

Coarse grained model development

Due to the absence of cross interaction parameters between the ionic liquids and lipids for the coarse grained simulations, a set of cross interaction had to be parameterized. For coarse grained simulations, the following Lorentz-Berthelot mixing rules

$$\varepsilon_{ij} = \sqrt{\varepsilon_{ii}\varepsilon_{jj}} \quad (1)$$

$$\sigma_{ij} = \frac{\sigma_{ii} + \sigma_{jj}}{2} \quad (2)$$

can often be inaccurate. Therefore, the cross parameters were instead fit to the PMF or relative free energy change for [C_4mim]$^+$ insertion into a bilayer obtained from our previous atomistic simulations.[1] For consistency, we used the same simulation conditions and free energy calculation method (umbrella sampling and the weighted histogram analysis method), as that of our previous

atomistic simulations to obtain the PMFs. As a starting point, we used the Lorentz-Berthelot mixing rules to estimate the cross interaction parameters, and scaled ε_{ij} and σ_{ij} such that:

$$\varepsilon_{ij,new} = \alpha \varepsilon_{ij,LB} \tag{3}$$

$$\sigma_{ij,new} = \alpha' \sigma_{ij,LB} \tag{4}$$

where $\varepsilon_{ij,LB}$ and $\sigma_{ij,LB}$ are the Lorentz-Berthelot parameters, α and α' are arbitrary adjustable parameters used to fit the relative free energy curve, and $\varepsilon_{ij,new}$ and $\sigma_{ij,new}$ are the parameters used in the bulk simulations. A simulation time of 40 ns for each umbrella sampling window was sufficient to obtain adequate free energy convergence. Note that coarse grain simulation time is typically faster than atomistic simulation time, thereby allowing faster convergence than compared to our previous atomistic simulation time of 80 ns per window.

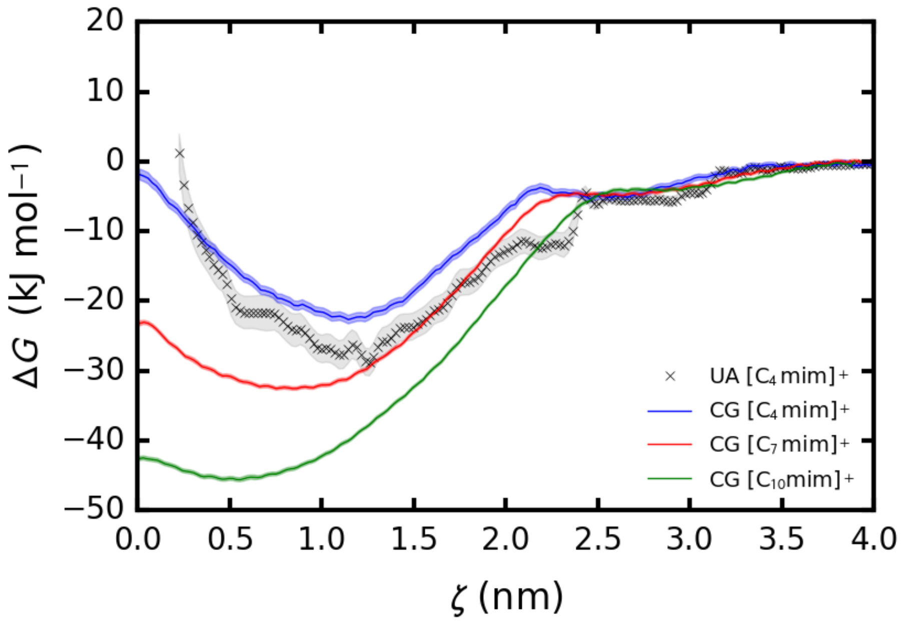

Figure S2 PMF mapping of coarse grained model cross interactions. Potential of mean force mapping of the cross interactions between a united atom and coarse grained models. The reaction coordinate is defined as the center of mass distance between the lipid bilayer and terminal alkyl group of the IL cation side chain. The transferability of these interaction parameters are shown by the larger decrease in free energy for increasing alkyl chain side chain in the cation, as well as the decrease in the location of each minimum. The dashed blue line is the PMF for $[C_4mim]^+$ insertion using standard Lorentz-Berthelot mixing rules. Uncertainties in the curves are represented by the shaded regions.

Figure S2 shows the free energy curves along a reaction coordinate (ζ), defined as the distance

between the bilayer center of mass in the Z-direction and the Z position of the terminal alkyl group of a IL cation alkyl chain. The coarse grained IL and POPC cross interaction $\sigma_{ij,new}$ and $\varepsilon_{ij,new}$ have been fitted to that of our previous atomistic simulations. A dielectric constant of ε_r = 80 was used during the parameterization. For the majority of the parameters, $\sigma_{ij,new}$ were unchanged from those obtained using the Lorentz-Berthelot mixing rules. For the $\varepsilon_{ij,new}$ parameters, the majority of them were determined by scaling the Lorentz-Berthelot parameters by a factor of α = 1.6 (deeper energy well), as the Lorentz-Berthelot mixing rules tended to under predict the magnitude in free energy minimum. For a few of the lipid headgroup and IL interaction parameters, α and α' was set to 0.25 and 1.25, respectively. As shown in Fig. S2, the location of the minimum for the free energy change and the magnitude in in free energy change for inserting $[C_4mim]^+$ into the POPC bilayer is in excellent agreement with that obtained from the atomistic simulations and also within the estimated uncertainties. The dashed line in the figure shows the corresponding PMF using the Lorentz-Bertholot mixing rules. Chloride anion and lipid cross interactions were obtained by using the same ε and σ parameters from water while correcting for its mass and charge, as has been done by Shinoda *et al.* for parameterization of sodium and chloride with water and SDS[2].

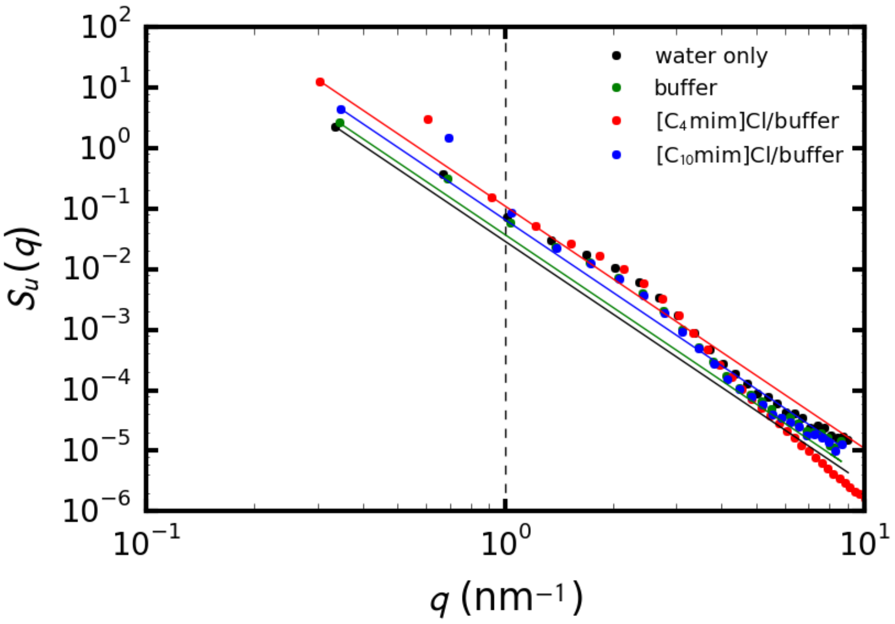

Figure S3 1D-undulation spectral intensity. Spectral intensity ($S_u(q)$) is plotted against wavenumber (q) for each of the studied (single) lipid bilayer systems: water only (black), buffer (green), [C$_4$mim]Cl/buffer, and [C$_{10}$mim]Cl/buffer. The corresponding solid lines are obtained by fitting the data for wavenumbers $q \leq$ 1.0 nm^{-1} (indicated by the dotted vertical line) to the Helfrich free energy model. Data shown is for the last 20 ns of each system, although the bending modulus reported is based on block averaging the data over the last 100 ns. Spectral intensities were obtained after taking the inverse Fourier transform of the bilayer undulation surface.

All bending moduli of the bilayer surface were determined using the Helfrich model[3]. The bilayer undulation surface were obtained by taking the phosphate head group coordinates for each lipids and spline-interpolating between each point onto a grid. Fast Fourier transforms of the undulation surface were performed to obtain the 2D power spectrum, and subsequently the 1-D spectral intensity plot (Fig. S3) used to determine the bending modulus. Each bending modulus has been block averaged from at least 100 ns trajectories.

References

[1] Yoo, B.; Shah, J. K.; Zhu, Y.; Maginn, E. J. *Soft Matter* **2014**, *10*, 8641–8651.

[2] Shinoda, W.; DeVane, R.; Klein, M. L. *Molecular Simulation* **2007**, *33*, 27–36.

[3] Helfrich, W. *Zeitschrift für Naturforschung. Teil C: Biochemie, Biophysik, Biologie, Virologie* **1973**, *28*, 693.
